# Comparative Efficacy of Intralesional Chloroquine With Intralesional Meglumine Antimoniate in the Treatment of Cutaneous Leishmaniasis

**DOI:** 10.7759/cureus.56785

**Published:** 2024-03-23

**Authors:** Obed Ullah, Muhammad Rizwan, Naeem Raza, Sumeera Zulfiqar, Nadia Akbar, Habib Ullah

**Affiliations:** 1 Department of Dermatology, Pak Emirates Military Hospital, Rawalpindi, PAK; 2 Department of Dermatology, Combined Military Hospital, Lahore, PAK; 3 Department of Dermatology, Sandeman Provincial Hospital, Quetta, PAK

**Keywords:** antimoniate, intralesional, chloroquine, efficacy, cutaneous leishmaniasis

## Abstract

Background and aim: This comparative prospective study was conducted at the Department of Dermatology, Pak Emirates Military Hospital, Rawalpindi, from August 1, 2018, to January 31, 2019 (six months). This study aimed to compare the efficacy of intralesional chloroquine with intralesional meglumine antimoniate in the treatment of cutaneous leishmaniasis.

Materials and methods: A total of 64 patients fulfilling the inclusion criteria reporting to the Department of Dermatology, Pak Emirates Military Hospital were included in this study. Informed consent was taken and demographic data including patients' hospital registration number, age, gender, and number of lesions were noted.

The subjects were randomly assigned into two groups. In group A, intralesional chloroquine was injected two times per week, and in group B, intralesional meglumine antimoniate was injected two times per week. The efficacy of both treatments was noted after eight weeks of treatment. Frequency and percentages were computed for qualitative variables like gender and number of lesions. Mean±standard deviation was presented for quantitative variables like age. Analysis was done to compare the proportion of both groups. Chi-square test was applied to compare the efficacy of both groups, p≤0.05 was taken as significant.

Results: In this study, the mean age of patients was 29.69±08.95 years. There were 63 (98.44%) males and one (1.56%) female. In this study, efficacy was achieved in six (18.8%) patients in group A, while in 17 (53.1%) patients in group B. This difference was statistically significant, i.e., p=0.004.

Conclusion: This study concluded that intralesional meglumine antimoniate is more effective in treating cutaneous leishmaniasis than intralesional chloroquine.

## Introduction

Leishmaniasis is an infectious disease caused by protozoa of the genus Leishmania, having a wide spectrum of clinical manifestations, depending on the involved species of Leishmania. Leishmaniasis was originally considered a wild zoonosis, but cases have been reported in domestic animals in rural and urban areas, especially in dogs [1]. There are about 30 species and subspecies of sand flies with documented vectors, and more than 40 additional species are also believed to be involved in the transmission of the disease [2].

Cutaneous leishmaniasis (CL) can present with a spectrum of clinical manifestations, the most common of which is ulcerative skin lesions occurring at the site of the bite of the sand fly. Cutaneous leishmaniasis remains disfiguring and stigmatizing and often heals with scarring. There are several more rare clinical manifestations like diffuse CL (DCL), which is usually difficult to treat [3].

Mucosal or mucocutaneous leishmaniasis is often a destructive form with mucosal inflammation, which has been reported mainly in the New World in association with *Leishmania braziliensis* but it is also reported in the Old World [4]. Worldwide, around one million cases of CL occur per year [5]. *Leishmania braziliensis *causes the largest CL burden in the New World, with Brazil being the most severely affected. In the Old World, cases are concentrated in North Africa, the Middle East, the Indian subcontinent, and Central Asia [5].

The first-line drug for the treatment of all forms of leishmaniasis is meglumine antimoniate [6,7]. Patients with CL lesions not responding to meglumine antimoniate treatment have been reported, and these patients may require alternative treatment options. A cross-sectional study showed that almost 88% of the patients responded to treatment with meglumine antimonite [6]. Intralesional meglumine antimoniate used in these days for the treatment of this parasitic disease is associated with high cost and resistance [8]. A study by Silva et al. has shown an efficacy of 73.3% with meglumine antimoniate in CL patients [9].

The search for an alternative agent has led to the fact that antimalarials (mefloquine, chloroquine, and hydroxychloroquine) are active against intracellular amastigotes in macrophage-infected cultures [8-10]. Chloroquine has appeared to be a well-tolerated and economical alternative to antimonials when given to the lesional area, and positive response was demonstrated with it in patients with CL. A study by Hanif et al. has shown an efficacy of 52% with intralesional chloroquine in the treatment of CL [11].

There is a paucity of data on this subject in our local population, moreover, no comparative study has been found so far in Pakistan that compares these two agents. It is clear that randomized control trials are highly needed and should be undertaken. This prompted us to compare the efficacy of intralesional chloroquine with meglumine antimoniate in the treatment of CL in our local population. The results of this study will help to select an appropriate alternative treatment option for the treatment of CL.

## Materials and methods

This randomized controlled trial was conducted at the Department of Dermatology, Pak Emirates Military Hospital Rawalpindi. The duration of this study was six months. The sampling technique used was consecutive (non-probability). The sample size was calculated with a 95% confidence level and alpha=5% (two-sided) with power=90%. Here, p1 represents the expected proportion (efficacy) in population 1 in the reference study, which is 52%, and p2 represents the expected proportion (efficacy) in population 2 in the reference study, which is 88% [10,11].

Group A comprised of patients who were treated with intralesional chloroquine, whereas group B comprised of patients who were treated with intralesional meglumine antimoniate. The sample size was calculated using the WHO sample size calculator, based on the results of the reference study [12]. The total sample size calculated was 64 patients. The power of the test was 90% with a level of significance of 5%.

Inclusion and exclusion criteria

Inclusion criteria were patients with an age range of 18-60 years of both genders and lesions not located on the face, neck, or joints on physical examination. Patients with more than three CL lesions with sizes more than 5 cm in diameter on physical examination, history of previous antileishmanial treatment on medical records, and sporotrichoid spread were excluded from this study.

Data collection

This study was conducted after getting approval from the hospital’s ethical and research committee having the reference number A/56. All patients meeting the inclusion criteria were included in the study through the outpatient department. The purpose and benefits of the study were explained to the patient and they were assured that the study was done purely for data publication and research purposes and written consent was taken. The demographic data including patients' hospital registration number, age, gender, and number of lesions were noted. The subjects were randomly assigned by blind balloting into one of the two groups.

In group A, intralesional chloroquine (Leverkusen, Germany: Bayer AG) was injected two times per week (with a three-day gap between two injections) within the lesions with a 30 G (0.3-8 mm) needle mounted on an insulin syringe. In group B, intralesional Glucantime (meglumine antimoniate) was injected two times per week (with a three-day gap between two injections) within the lesions with a 30 G (0.3-8 mm) needle mounted on an insulin syringe. The dose of injections used was 0.5-1 cc/cm^2^ of the lesion. The efficacy of both treatments was noted after eight weeks of treatment as per operational definition and recorded on a specially designed proforma.

Data analysis

Data were analyzed with the statistical analysis program, SPSS version 22 (Armonk, NY: IBM Corp.). Frequency and percentages were computed for qualitative variables like gender and number of lesions. The mean±standard deviation was presented for quantitative variables like age. Analysis was done to compare the proportion of both groups. Chi-square test was applied to compare the efficacy of both groups, p≤0.05 was taken as significant.

Stratification was done with regard to age, gender, and number of lesions to see the effect of these variables on efficacy. Post-stratification chi-square test for both groups was applied, and p≤0.05 was considered statistically significant.

## Results

In our study the mean age of group A patients was 31.13±09.88 years whereas in group B patients was 28.31±07.59 years. In group A, there were 31 (96.9%) males and one (3.1%) female. In group B, there were 32 (100%) males and no (0.0%) females as shown in Table 1. In group A, 14 (43.8%) patients had one lesion, while 18 (56.2%) had two lesions. In group B, 14 (43.8%) patients had one lesion, while 18 (56.2%) had two lesions as shown in Table 2.

**Table 1:** Frequency distribution of genders in both groups.

Variables		Group A (n=32)		Group B (n=32)		Total (n=64)	
		No. of patients	Age (%)	No. of patients	Age (%)	No. of patients	Age (%)
Gender	Male	31	96.9	32	100	63	98.4
	Female	01	3.1	00	0.0	01	1.6
Total		32	100	32	100	64	100

**Table 2:** Frequency distribution of number of lesions in both groups.

Variables		Group A (n=32)		Group B (n=32)		Total (n=64)	
		No. of patients	Age (%)	No. of patients	Age (%)	No. of patients	Age (%)
Number of lesions	One	14	43.8	14	43.8	28	43.8
	Two	18	56.2	18	56.2	36	56.2
Total		32	100	32	100	64	100

**Figure 1:**
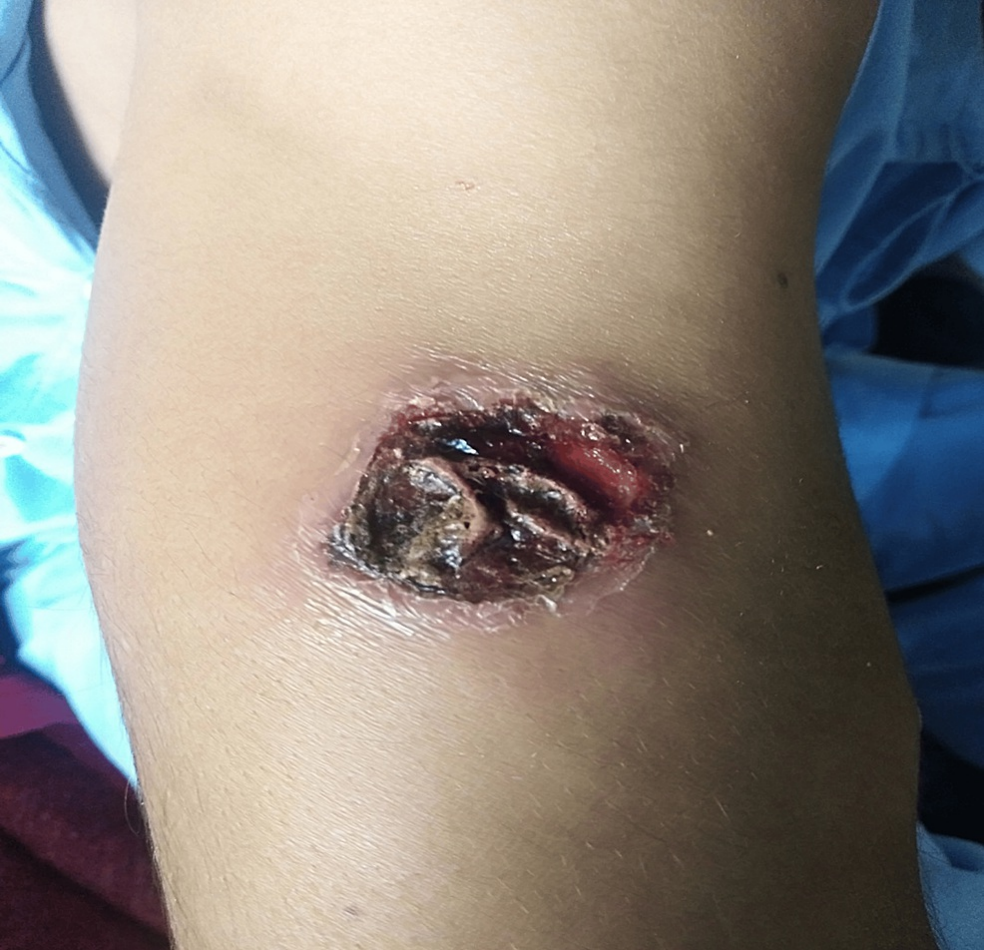
Group A patient before treatment. Site: right arm

**Figure 2:**
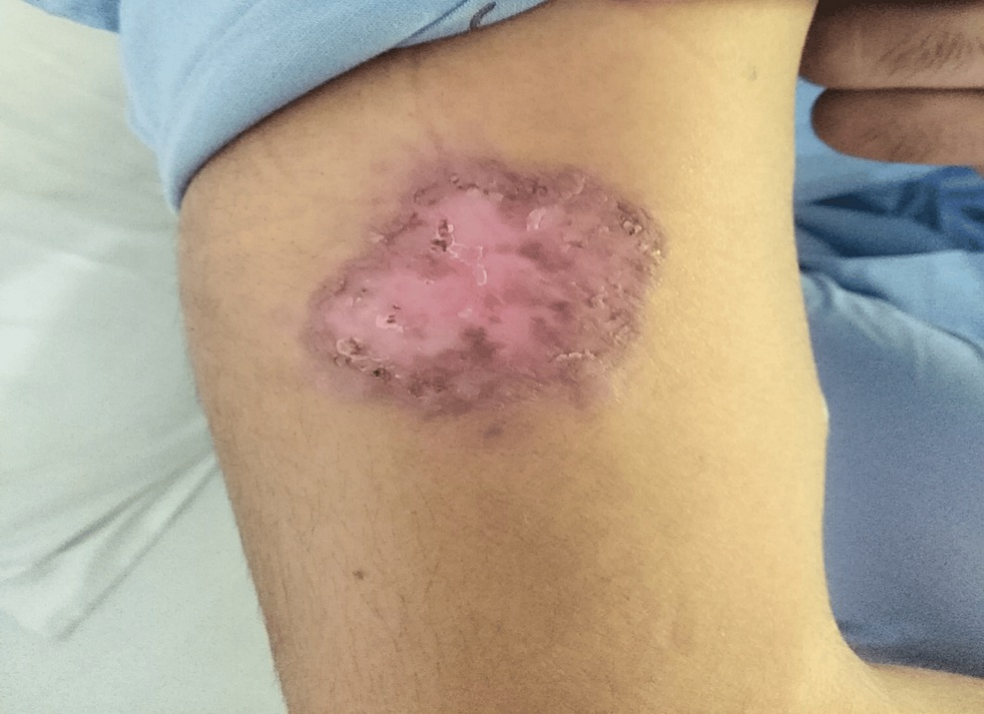
Group A patient after treatment. Site: right arm

**Figure 3:**
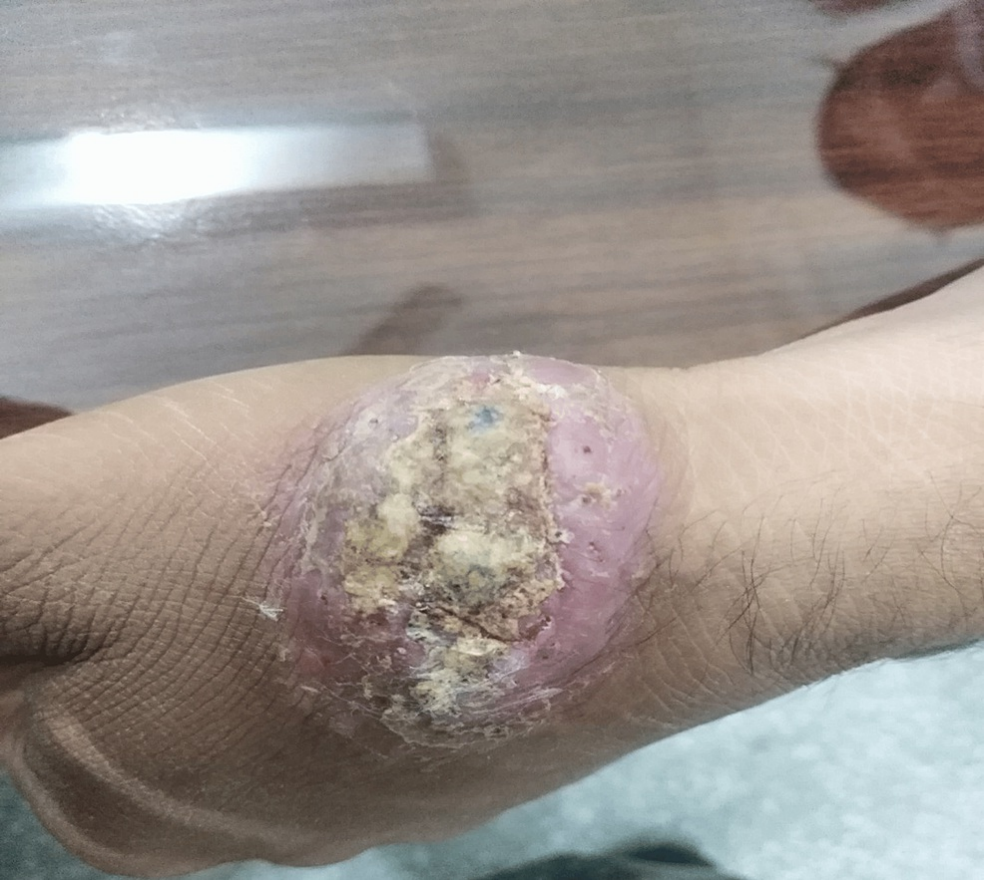
Group B patient before treatment. Site of infection: left hand

**Figure 4:**
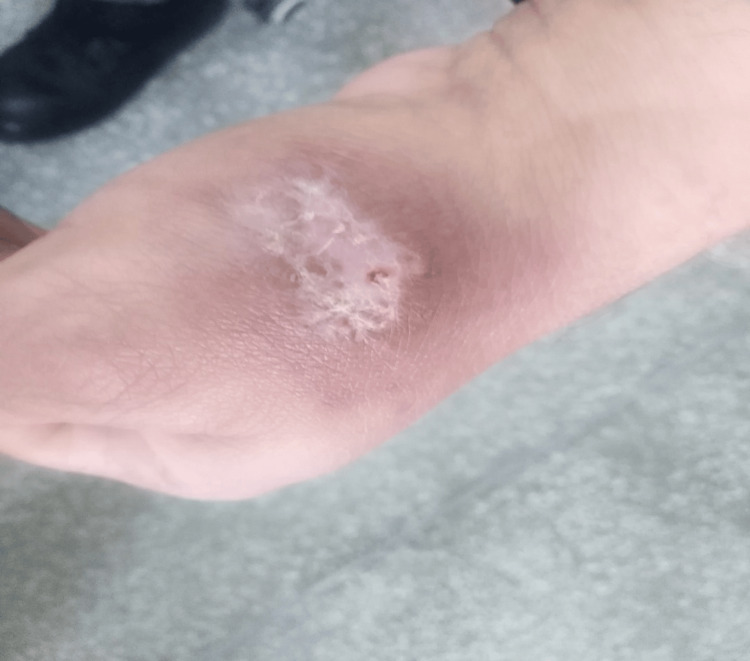
Group B patient after treatment. Site of infection: left hand

In this study, efficacy was achieved in six (18.8%) patients in group A (Figures 1, 2). While in 17 (53.1%) patients in group B, after completion of treatment (Figures 3, 4). This difference was statistically significant, i.e., p=0.004 (Table 3). Data was stratified for the number of lesions. In patients who had one lesion, efficacy was achieved in five (35.7%) cases in group A, while in 10 (71.4%) cases in group B. The difference was insignificant (p>0.05). In patients who had two lesions, efficacy was achieved in one (5.6%) case in group A, while in seven (38.9%) cases in group B. The difference was significant (p<0.05) (Table 4).

**Table 3:** Comparison of efficacy achieved in both groups.

Variables		Group A (n=32)		Group B (n=32)		Total (n=64)	
		No. of patients	Age (%)	No. of patients	Age (%)	No. of patients	Age (%)
Efficacy	Yes	6	18.8	17	53.1	32	35.9
	No	26	81.3	15	46.9	41	64.1
Total		32	100	32	100	64	100

**Table 4:** Comparison of efficacy achieved in both groups stratified by number of lesions.

No. of lesion	Efficacy	Group A (n=32)	Group B (n=32)	Total (n=64)	p-Value
One	Yes	5 (35.7%)	10 (71.4%)	15 (53.6%)	0.058
	No	9 (64.3%)	4 (28.6%)	13 (46.4%)	
	Total	14 (100%)	14 (100%)	28 (100%)	
Two	Yes	1 (5.6%)	7 (38.9%)	8 (22.2%)	0.016
	No	17 (94.4%)	11 (61.1%)	28 (77.8%)	
	Total	18 (100%)	18 (100%)	36 (100%)	

## Discussion

CL is a major health problem worldwide. It is also a particular problem in the rural areas of Pakistan. Several species of the genus Leishmania are involved in its causation and infection is transmitted by the bite of a sand fly. Chloroquine has appeared to be a well-tolerated and economical alternative to antimonials when given into the lesional skin with a positive response demonstrated in patients with CL. It is a recent addition to the treatment of CL [11]. In addition to its easy availability, chloroquine has the benefit of intralesional administration and low cost compared to antimonials, which is of particular significance in developing countries including Pakistan [13]. Moreover, there are many patients who have contraindications to antimonials, for whom other treatment modalities need to be developed [14].

A study by Hanif et al. in which they compared intralesional chloroquine with oral chloroquine, documented that the cure rate was 100% in both groups towards the end of treatment. The mean duration of treatment was 9.17 weeks in intralesional chloroquine as against 11.37 weeks in oral chloroquine (p=0.0028). The mean total dose of the drug given to each patient in intralesional chloroquine was 5.8±0.5 g and for oral chloroquine, it was 19.2±1.5 g, which is significantly higher (p<0.001).

In a previous study conducted on the treatment of CL, intralesional chloroquine was compared with intralesional meglumine antimoniate and was found to be well tolerated, more economical, and easily available alternative to antimonials; and patients showed equally good response [14]. One study by Yasmin et al. revealed that both treatments showed 100% response; however, a greater number of injections were required with meglumine antimoniate (p<0.05) [15].

In our study, we stratified data for the age of patients to check the impact of age on the efficacy of trial drugs. It was observed that intralesional meglumine antimoniate was significantly more effective (13 {61.9%}) in patients aged ≤35 years than intralesional chloroquine (3 {12.5%} cases) (p<0.05). While the efficacy with both treatment drugs was almost equal or in other words, statistically insignificant in patients aged >35 years, i.e., three (37.5%) cases with intralesional chloroquine vs. four (36.4%) with intralesional meglumine antimoniate (p>0.05).

Data was stratified for the gender of patients. Among males, efficacy was significantly higher with the use of intralesional meglumine antimoniate (17 {53.1%} cases) as compared to intralesional chloroquine (6 {19.4%} cases) (p<0.05). However, calculations could not be done in females as there was a single female patient and she was randomized to intralesional chloroquine and achieved efficacy.

Regarding the side effect profile, there was no significant side effect noted except for local injection site pain and transient swelling. Among these groups, pain was noted more in the intralesional chloroquine group compared to the intralesional meglumine antimoniate group.

We stratified the data according to the number of lesions. In patients who had one lesion, intralesional chloroquine was efficacious in five (35.7%) cases, while in patients receiving intralesional meglumine antimoniate, 10 (71.4%) cases were treated effectively. The difference was insignificant (p>0.05). In patients who had two lesions, efficacy was achieved in one (5.6%) case with intralesional chloroquine and seven (38.9%) cases with intralesional meglumine antimoniate (p<0.05). So in cases with >1 lesion, intralesional meglumine antimoniate is found to be more effective than intralesional chloroquine. So intralesional meglumine antimoniate for management of CL may be considered an option.

Study limitations

The sample size of the study was very small, so there is a need to conduct further trials on large sample sizes as well as responses to different doses of intralesional meglumine antimoniate need to be researched further.

## Conclusions

Our study result showed that the efficacy of the intralesional meglumine antimoniate group was considerably higher as compared to intralesional chloroquine. Not much literature is available on this topic, so it is suggested that in future further studies be conducted with more sample size to validate the findings of our study. The sample size of our study was very small, so there is a need to conduct further trials on large sample sizes as well as responses to different doses of intralesional meglumine antimoniate need to be researched further. Furthermore, in resource-poor countries where availability of meglumine antimoniate is a major problem, intralesional chloroquine can be used as second-line treatment.
